# Long-term quantification and brief-pulse optogenetic perturbation of nucleocytoplasmic GtaC dynamics during Dictyostelium development

**DOI:** 10.17912/micropub.biology.002037

**Published:** 2026-04-06

**Authors:** Kensuke Yamashita, Tetsuya Muramoto

**Affiliations:** 1 Department of Biology, Faculty of Science, Toho University

## Abstract

Transcription factor nucleocytoplasmic dynamics play a key role in developmental gene regulation. In *Dictyostelium* , the transcription factor GtaC exhibits nucleocytoplasmic shuttling, but its shuttling trajectory across multicellular aggregation has not been systematically quantified. Using a knock-in strain, we tracked GtaC dynamics from starvation through aggregation and quantified developmental changes in shuttling period, amplitude, and synchrony. Notably, brief-pulse optogenetic activation of cAMP at higher input frequencies reproduced the reported attenuation of GtaC shuttling amplitude with high temporal precision and minimal phototoxicity. Together, long-term quantification and brief-pulse optogenetic cAMP perturbation show that GtaC nucleocytoplasmic shuttling is developmentally tuned in a frequency-dependent manner.

**Figure 1. Developmental dynamics of endogenous GtaC nucleocytoplasmic shuttling:** **A)** Subcellular localization of endogenously tagged mNG-GtaC during early developmental stages. Developmental time after starvation is indicated above each image. Representative images are shown. Scale bars: 50 µm. See Movie 1 for time-lapse imaging. **B)** Time-series analysis of endogenous GtaC nucleocytoplasmic dynamics. The nuclear-to-cytoplasmic (N/C) fluorescence ratio was quantified for individual cells aligned according to developmental time. Solid lines represent the mean N/C ratios across cells within the representative field of view shown, and the shaded areas indicate ± SD (n = 60–85 cells). In total, 12–16 fields of view from at least three independent experiments were analyzed for each developmental time point. **C)** Quantification of oscillatory properties of endogenous GtaC dynamics during development. Period and amplitude were calculated from time-series data of the N/C ratio using wavelet-based analysis, and a synchrony index was quantified using the Kuramoto order parameter. Box plots show the distribution of per-field means at each developmental time point, with individual fields overlaid as colored points. Data were obtained from 12–16 fields of view per time point. **D)** Modulation of GtaC shuttling dynamics by optogenetic control of the cAMP oscillation frequency. Cells expressing endogenously tagged mScarlet-I-GtaC and the photoactivated adenylyl cyclase mPAC were stimulated with brief 8-s pulses of blue light at 3–4 h of development. The stimulation period was modulated from 5 min to 2 min in 1-min increments, and N/C dynamics were quantified. Solid red lines represent mean N/C ratios and error bars indicate ± SD. Data were collected from 128–144 cells under constant 5-min stimulation and from 154–317 cells under frequency-modulated stimulation conditions. For each condition, three independent experiments were performed, and a representative dataset is shown.

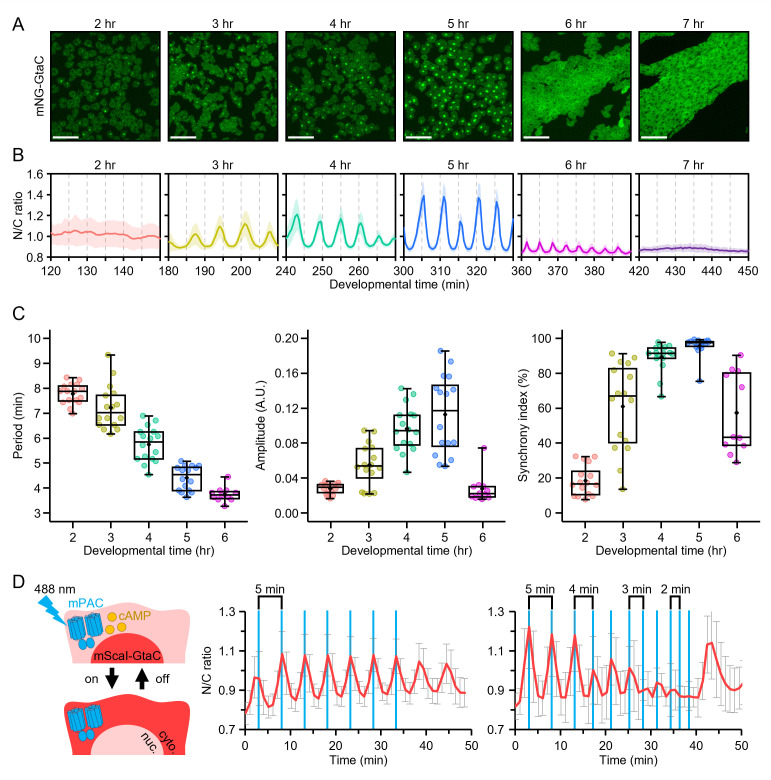



## Description

Cells can encode extracellular cues as temporal patterns of signaling activity that downstream effectors decode through time-varying dynamics to guide cellular decision-making (Behar and Hoffmann 2010; Bonsignore et al. 2025; Purvis and Lahav 2013). Stimulus identity and context can be embedded in these dynamics and mapped to distinct outcomes, as shown for ERK/MAPK activity patterns (Ram et al. 2023; Ryu et al. 2015; Wilson et al. 2017) and for NF-κB nuclear localization programs that shape gene regulation (Maity and Wollman 2020; Martin et al. 2020; Wibisana and Okada 2022). Such dynamics are commonly quantified by tracking post-translationally regulated changes in subcellular localization and summarizing trajectories with features such as duration, frequency, amplitude, and delay (Cai et al. 2008; Regot et al. 2014; Shrum et al. 2009). Combined measurements and targeted perturbations, together with quantitative and computational frameworks, enable systematic extraction, comparison, and interpretation of dynamic features across signaling conditions (Bennett et al. 2024; Kamino et al. 2017; Micali et al. 2015; Rosen et al. 2026).

In *Dictyostelium discoideum* , the transition from unicellular to multicellular development following nutrient deprivation involves the nucleocytoplasmic shuttling of the transcription factor GtaC, which plays a role in developmental gene expression (Cai et al. 2014). The shuttling of GtaC is responsive to the temporal structure of cAMP signaling, including pulsatile inputs, and is driven by phosphorylation of GtaC by the atypical MAPK Erk2 (Hadwiger et al. 2022). Furthermore, live imaging has quantified developmental changes in cAMP wave dynamics, particularly shifts in wave frequency and propagation patterns (Ford et al. 2023; Gregor et al. 2010; Hashimura et al. 2019; Singer et al. 2019). However, corresponding changes in the quantitative features of GtaC shuttling, such as period, amplitude, and synchrony, have not been systematically measured. A significant challenge has been the reliance on overexpression-based reporters, which can disrupt development and introduce localization artifacts, thereby obscuring endogenous GtaC dynamics over extended time periods. Recent advancements in CRISPR/Cas9-mediated genome editing now facilitate knock-in strategies to visualize endogenous GtaC (Yamashita et al. 2021; Yamashita and Muramoto 2025). Despite these advances, previous analyses of endogenous GtaC focused on developmental stages when shuttling is prominent (Yamashita and Muramoto 2025), and therefore did not systematically define its onset. Meanwhile, attenuation during later stages was measured (Yamashita et al. 2025), but a unified quantification of the GtaC shuttling trajectory during multicellular aggregation using the same experimental and analytical framework has not been reported. In this study, we perform long-term measurements of GtaC dynamics from early starvation responses through streaming to map the onset-to-termination trajectory of shuttling and quantify how its dynamics evolve over time. Utilizing these shuttling dynamics as a quantitative readout, we optimized stimulation conditions for our established optogenetic perturbation (Yamashita et al. 2025), enabling more precise, stepwise tuning of cAMP oscillation frequency while minimizing cellular phototoxicity.

To extend the developmental time course examined for GtaC localization, we performed time-lapse imaging of endogenously expressed, fluorescently tagged GtaC in knock-in cells from 2 to 7 hours post-starvation ( Fig. 1A ). This imaging period encompasses early development through the initiation of collective migration and subsequent streaming, allowing for direct comparison of GtaC localization states across distinct developmental stages under consistent imaging conditions. We quantified GtaC nuclear localization using the nuclear-to-cytoplasmic (N/C) ratio and observed a developmental shift in population-level coordination ( Fig. 1B ). At 2 hours, only a few cells exhibited sustained nuclear GtaC, and coherent periodic shuttling was not evident. In contrast, beginning at 3 hours, synchronized oscillatory shuttling emerged across the population, indicating the onset of coordinated, collective GtaC dynamics. The characteristics of these oscillations were strongly stage-dependent ( Fig. 1C ). Oscillatory behavior became most prominent at 5 hours, when shuttling amplitude and synchrony were maximal. As development progressed to 6 hours, coinciding with the onset of collective migration, oscillation amplitude decreased markedly. By 7 hours, when cell–cell adhesion increased and streaming structures formed, GtaC localization dynamics were significantly reduced and became biased toward the cytoplasmic state. These stage-dependent changes were consistently supported by quantitative analysis of oscillation period, amplitude, and synchrony across independent observations ( Fig. 1C ).

To investigate how gradual changes in GtaC shuttling characteristics interpret the temporal structure of upstream inputs, we used optogenetic manipulation of cAMP signaling. Conventional methods for modulating cAMP signaling, such as microinjection, caged compounds, and microfluidic systems, have yielded valuable insights but offer limited flexibility for continuous, within-experiment modulation of oscillation frequency &nbsp;(Nakajima et al. 2014; Rietdorf et al. 1998; Westendorf et al. 2013). In prior optogenetic studies, frequency modulation was achieved only coarsely, for example by changing the stimulation period from 6 to 4 min, a limitation often associated with 50% duty-cycle square-wave protocols intended to equalize total light exposure across periods (Yamashita et al. 2025). Moreover, under a fixed 50% duty cycle, stimulation duration necessarily scales with cycle length, leading to prolonged stimulation at longer periods and deviating from pulsatile cAMP signaling observed under physiological conditions. This prolonged stimulation could also trigger excessive or sustained cAMP production via endogenous ACA activity, complicating the interpretation of frequency-dependent responses. In this study, we therefore used a pulse-like input design in which a single fixed 8 s light pulse was delivered once per cycle, enabling seamless within-experiment frequency adjustments. In our previous work, we confirmed that intracellular cAMP increased to detectable levels with a 1-s light pulse and exceeded physiological synthesis levels with a 10-s pulse (Yamashita et al. 2025). Based on these results, we adopted an 8-s brief pulse as the shortest stimulus duration that reliably elicits a sufficient cAMP response. This protocol consistently elicited a localization response across independent experiments at 3–4 h of development, while avoiding prolonged stimulation within each cycle and thereby minimizing unnecessary light exposure, phototoxicity, and baseline elevation. As a reference, under a constant 5 min stimulation period, GtaC exhibited robust, stimulus-locked periodic shuttling ( Fig. 1D ). Reducing the stimulation period in 1 min increments decreased shuttling amplitude at a 4 min period and disrupted shuttling at a 3 min period, consistent with our previous findings (Yamashita et al. 2025). This stepwise fine-tuning of stimulation timing enabled frequency modulation of the optogenetically driven cAMP input at higher temporal resolution and long-term quantification of GtaC responses.

A significant advance of this study is the systematic quantification of GtaC shuttling across an extensive developmental window, rather than focusing only on stages where shuttling is prominent. This approach enabled accurate and reproducible measurements of stage-dependent variations in the period, amplitude, and synchrony of endogenous GtaC shuttling, which corresponded to population states ranging from localized coordination to collective migration and streaming. Additionally, brief-pulse optogenetic perturbation, implemented with high temporal precision, confirmed the frequency-dependent decrease in GtaC shuttling amplitude with minimal phototoxicity, consistent with our previous findings (Yamashita et al. 2025). Beyond GtaC, the capacity to measure and manipulate endogenous nucleocytoplasmic dynamics over prolonged developmental timescales offers a framework for comparative analysis of multiple transcription factors, including those such as Hbx5 (Brimson et al. 2025; Hao et al. 2024) that display developmentally regulated localization dynamics. Such comparisons may elucidate how distinct transcription factors interpret temporal features of cAMP signaling and coordinate gene expression programs across successive developmental stages. Collectively, these approaches connect endogenous GtaC dynamics across an extended developmental window to defined cAMP input patterns and provide a quantitative foundation for understanding how transcription factor response trajectories evolve across collective developmental states.

## Methods

**Cell lines and growth conditions**

*Dictyostelium discoideum* AX3–derived knock-in (KI) strains were maintained at 22°C in HL5 medium (Formedium) supplemented with streptomycin. mNeonGreen-tagged GtaC knock-in cells (mNG-GtaC KI; dTM1588) were used for live imaging (Yamashita and Muramoto 2025). For optogenetic experiments, the photoactivatable adenylyl cyclase mPAC was introduced into mScarlet-I-tagged GtaC knock-in cells (mScaI-GtaC KI; dTM2135) to generate mScaI-GtaC KI/mPAC cells (dTM2151) (Yamashita et al. 2025). To minimize physiological variability, cultures were used within 2 weeks after thawing from frozen stocks.

**Cell preparation for live imaging and optogenetic manipulation**

Cells were harvested during log-phase growth (1–4×10 ^6^ cells/mL), washed in KK2 phosphate buffer (16.5 mM KH _2_ PO _4_ , 3.9 mM K _2_ HPO _4_ ), and starved to initiate development. The time of washing was defined as 0 h of development. Cells were plated on agar at a density of 2.6×10 ^5^ cells/cm ^2^ in plastic dishes containing 1.5% agar (Difco Bacto-agar) prepared in KK2 buffer. After 10 min of incubation, excess KK2 buffer was carefully removed, and dishes were incubated at 22°C until the desired developmental time point. Approximately 30 min prior to imaging, agar was cut into 1-cm squares, and the agar surface with adherent cells was placed face down in glass-bottomed dishes (Delta T Culture Dishes, Bioptechs). Mineral oil (M8410, Sigma-Aldrich) was layered over the agar to prevent drying.

**&nbsp;Imaging and optogenetic conditions**

Live-cell imaging was performed on an inverted confocal microscope (Eclipse Ti-A1R, Nikon) equipped with a Plan Apo λ 60×/1.40 NA oil-immersion objective. Fluorescent tags were excited using solid-state lasers at 488 nm (mNeonGreen), 561 nm (mScarlet-I and mCherry), and 640 nm (miRFP670). mCherry- and miRFP670-tagged H2Bv3 signals were acquired for nuclear segmentation and cell tracking. To visualize GtaC shuttling, time-lapse images were acquired every 30 s as Z-stacks of 7 planes spaced 2 µm apart to capture nuclear and cytoplasmic signals, using the resonant scanner and a piezo Z stage (Nano-Drive, Mad City Labs). Each 30-min session included four fields of view per agar square. During optogenetic experiments, imaging was performed with a galvanometric scanner, and 488-nm light pulses were delivered between image acquisitions. A square region covering the entire field (0.21 mm × 0.21 mm) was illuminated at an intensity of 36.3 µW/µm ^2^ , and each light pulse lasted 8 s. For frequency-modulation experiments, the stimulation period was stepped from 5 to 2 min in 1-min increments, whereas control experiments used 8-s pulses at a constant 5-min period.

**Imaging analysis**

Time-lapse images were analyzed in Volocity (version 6.3, PerkinElmer) to quantify single-cell GtaC localization dynamics. Nuclear masks were segmented from the nuclear-marker channel, and the cytoplasmic regions were defined by isotropic expansion of each nuclear mask followed by subtraction of the nuclear region. Nuclear and cytoplasmic fluorescence intensities were measured over time to compute N/C ratio traces, and cells that left the field of view or could not be reliably tracked were excluded. Instantaneous period, amplitude, and phase were extracted by wavelet analysis using pyBOAT v0.9.8 (Schmal et al. 2022) running in Anaconda Navigator v2.2.0, enabling analysis of non-stationary oscillatory dynamics. Before the wavelet transform, trends were removed using a sinc filter implemented in pyBOAT. Because wavelet estimates near the edges of the time series are unreliable, time points overlapping the cone of influence (COI) were excluded from downstream quantification. Accordingly, the first and last 7.5 min (15 frames each) of each 30-min time-lapse were discarded from quantitative analyses. To quantify synchrony across cells within each field of view, a synchrony index was calculated based on the Kuramoto order parameter, which ranges from 0 (no synchrony) to 1 (perfect synchrony), using instantaneous phase information (Acebrón et al. 2005). Calculations were performed in R (v4.3.0) with the circular package by computing the mean resultant length at each time point with rho.circular function, followed by time-averaging across the imaging period.

## Reagents

**Plasmids**

**Table:** 

**Plasmid ID**	**Plasmid name**	**Description**	**Drug Resistance**	**Reference**
pTM2559	[coaA]: mPAC	Extrachromosomal vector for mPAC expression under the *coaA* promoter	Hygromycin B	(Yamashita et al. 2025)

**Cell lines**

**Table:** 

**Strain ID**	**Description**	**Parental Strain**	**Reference**
dTM1482	mCherry-H2B Knock-in	Ax3	(Yamashita and Muramoto 2025)
dTM1588	mNeonGreen-GtaC Knock-in	dTM1482	(Yamashita and Muramoto 2025)
dTM2015	miRFP670-H2B Knock-in	Ax3	(Yamashita et al. 2025)
dTM2135	mScarlet-I-GtaC Knock-in	dTM2015	(Yamashita et al. 2025)
dTM2151	mScarlet-I-GtaC Knock-in/mPAC	dTM2135	(Yamashita et al. 2025)

## Data Availability

Description: Time-lapse movie of endogenous GtaC nucleocytoplasmic shuttling dynamics from early starvation responses through the streaming stage. Images were acquired every 30 s for 30 min at each developmental time point. Scale bars: 50 µm.. Resource Type: Audiovisual. DOI: https://doi.org/10.22002/yag9s-rjd92
